# Evaluation of a novel nanocrystalline hydroxyapatite paste Ostim^® ^in comparison to Alpha-BSM^® ^- more bone ingrowth inside the implanted material with Ostim^® ^compared to Alpha BSM^®^

**DOI:** 10.1186/1471-2474-10-164

**Published:** 2009-12-22

**Authors:** Franz-Xaver Huber, Nicholas McArthur, Lydia Heimann, Elvira Dingeldein, Héloïse Cavey, Xavier Palazzi, Gaëlle Clermont, Jean-Pierre Boutrand

**Affiliations:** 1 Klinik für Unfallchirurgie, Orthopädie und Wiederherstellungschirurgie, Klinikum Ansbach, Escherichstraße 1 91522 Ansbach, Germany; 2 Medway Maritime Hospital, Gillingham, Kent, ME7 5 NY, UK; 3 aap bio implants group, Industrie Center Obernburg, D - 63784 Obernburg, Germany; 4 BIOMATECH-NAMSA, Zone Industrielle de I'Islon, 115 Rue Pasteur, F - 38670 Chasse-sur-Rhone, France

## Abstract

**Background:**

The purpose of this study was to evaluate the performance a newly developed nanocrystalline hydroxyapatite, OSTIM^® ^following functional implantation in femoral sites in thirty-eight sheep for 1, 2 or 3 months. Ostim^® ^35 was compared to an established calcium phosphate, Alpha BSM^®^.

**Methods:**

Biomechanical testing, μ-CT analysis, histological and histomorphological analyses were conducted to compare the treatments including evaluation of bone regeneration level, material degradation, implant biomechanical characteristics.

**Results:**

The micro-computed tomography (μCT) analysis and macroscopic observations showed that Ostim^® ^seemed to diffuse easily particularly when the defects were created in a cancellous bone area. Alpha BSM^® ^remained in the defect.

The performance of Ostim was good in terms of mechanical properties that were similar to Alpha BSM^® ^and the histological analysis showed that the bone regeneration was better with Ostim^® ^than with Alpha BSM^®^. The histomorphometric analysis confirmed the qualitative analysis and showed more bone ingrowth inside the implanted material with Ostim^® ^when compared to Alpha BSM ^® ^at all time points.

**Conclusions:**

The successful bone healing with osseous consolidation verifies the importance of the nanocrystalline hydroxyapatite in the treatment of metaphyseal osseous volume defects in the metaphyseal spongiosa.

## Background

Operative reconstruction of bone defects beyond a certain size still remains a challenge to trauma and orthopedic surgeons. Every year, millions of people worldwide are suffering from bone defects arising from trauma, tumor or bone diseases. In approximately 10% of all traumatically related loss of bone structure or even tumor related bone defects, spontaneous bone healing is not able to restore the required physiological stability. In such cases bone replacement materials are often necessary to reconstruct the anatomical morphology and restore stability of the bone[1].

The use of autologous pelvic bone is still considered as the gold standard in the reconstruction of bone defects because of its unsurpassed biological activity even in implant sites with low osteogenic potential. Pelvic bone harvesting from the iliac crest does, however, presents unacceptable rates of morbidity at the grafting site and at the same time may only provide a limited amount of cancellous bone[2-8]. Chronic pain can be present in up to 39% of patients at the donor site after iliac crest harvesting[2]. Other published complications include: fractures, infection, nerve and arterial injury[7].

Other bone sources include bone allografts which carry the potential of disease transmission, immunogenicity and possibly lower union rates[4,9]. Furthermore, the structural, mechanical, and resorption properties of allografts are usually much altered by processing, preservation, and sterilization techniques[4,10,11]. The relative concerns over the use of either autograft or allograft have led to the development of numerous bone graft substitutes[12-20].

In the ideal case artificial bone replacement materials should present a similar structure and composition to human bone and thus be able to present bone function. The materials should be osteoconductive and osteoinductive by allowing osteoblast and osteoclast activity. At present there are over 100 approved bone replacement materials in Germany alone. The spectrum encompasses mainly hydroxyapatite ceramics, absorbable calcium phosphate cements, various metals, plastics and a variety of composites. The most commonly used synthetic mineral substitutes for bone defect and trauma applications as implant coatings and defect fillers are hydroxyapatite cements, which have already undergone comprehensive animal testing and have also established themselves in many surgical procedures on human patients[18-39].

Ostim^® ^represents a brand new development among the purely synthetically produced and rapidly absorbable Hydroxyapatite compounds. It has been widely and successfully used in the fields of oral and maxillofacial surgery and orthopedic and trauma surgery[40-46].

The aim of the following study was to compare the newly developed Ostim^® ^with tricalcium phosphate cement Alpha-BSM^®^, an already established bone replacement material, in relation to their biocompatibility and bone ingrowth in a bone defect.

## Methods

### Material properties of the hydroxyapatite compounds used

#### Ostim^®^

**Figure 1:**
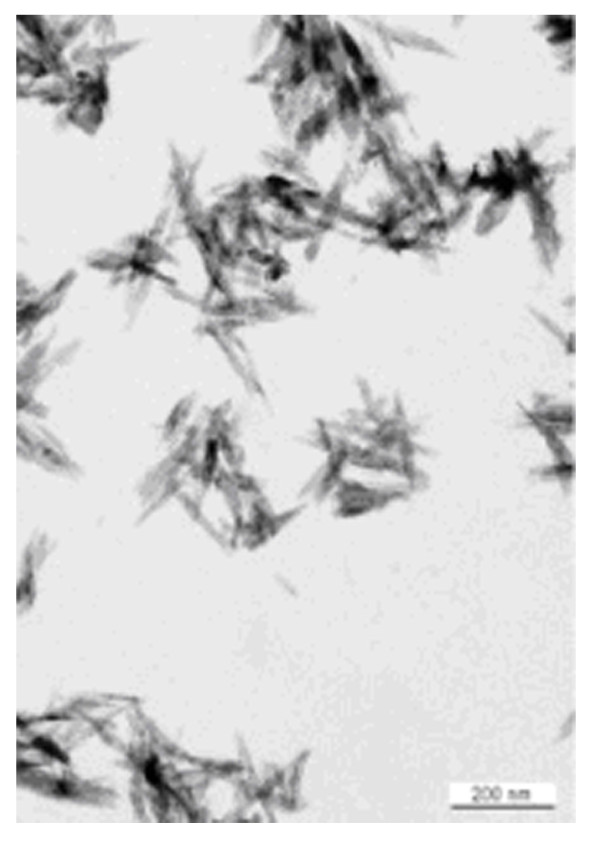
**Sample dimensions**. The test specimens were prepared so that the implantation site was in the centre of the sample. The tissue samples were provided in physiological serum and the trials were performed within 48 h after removal.

Ostim^®^, (aap biomaterials GmbH, Dieburg, Germany) is a newly developed, fully synthetic and fully resorbable injectable nanocrystalline paste [Ca10(PO4)_6_(OH)_2_] and consists of a suspension of pure HA in water prepared by a wet chemical reaction. The needle shaped HA crystals form agglomerates as shown by transmission electron microscopy (see fig. 1). XRD Analysis reveals an average crystallite size of 19 nm. Ostim^® ^paste does not harden after application into the bone and is free of endothermal heating. It is characterized by a large bioactive specific surface area of 106 m^2 ^g-^1^[47]. The atomic ratio of calcium-phosphorus is 1.67.

The product is supplied in a ready-to-use syringe to which a needle or a flexible 5 cm nozzle can be attached in order to inject the paste into deeper voids.

#### Alpha BSM^®^

Alpha BSM^® ^(ETEX Corp, CAMBRIDGE, MA 02139, USA), an established calcium phosphate cement, is an endothermically setting apatitic calcium phosphate bone substitute. The final product (means after the conversion reaction is complete) consists of purely crystalline HA with an average crystallite size of 12-14 nm. Alpha BSM^® ^has a paste like consistency until it is injected and hardens at body temperature. In 20 min it achieves complete conversion and mechanical strength. The reported mechanical strength is 15 MPa[34]. Specific surface area of the dried product is 78 m^²^/g.

None of two materials has macropores and both are applied as wet pastes. The water content of Ostim is 65%, freshly prepared alpha-BSM paste has a water content of 55%.

### Animal experiment protocol

Permission was granted to conduct all parts of our animal experiments we applied for (authorization number 59036 by BIOMATECH Ethical Committee on March 16, 2007). The sheep model has been used in intra-osseous implantation studies and is recognized by International Regulatory organizations[48,49]. Thirty eight with three in reserve (38 + 3) female sheep (Ovisaries), between 53 and 73 kg and between 2 and 5 years old were used for the animal experiments.

The evaluation of the performance and the biocompatibility of a bone substitute was tested following functional implantation in femoral sites in thirty-eight sheep for 1, 2 or 3 months (total of 76 sites). The Ostim^® ^material (n = 38 sites) was compared to Alpha-BSM^® ^(n = 38 sites) following implantation in a cancellous bone defect (see additional file 1).

Each animal received an implantation of each bone replacement material in the cancellous bone of the right and left medial distal femoral epiphysis respectively. The treatment allocation was randomly assigned between the right and left leg. Pre-medication and anesthesia were performed by intravenous injection of thiopental-pentobarbital mixture (NESDONAL^®^, MERIAL, France; pentobarbital sodique, CEVA Santé animale, France) and atropine (Atropinum sulfuricum, AGUETTANT, France) followed by inhalation of a O2 - isoflurane (1-4%) mixture. Each animal received an analgesic (flunixine, FINADYNE^® ^Injectable, Schering-Plough, France) pre-operatively. As a prophylactic measure, perioperative antibiotics penicillin-procaine and penicillin benzathine (DUPHAPEN^® ^LA, FORT DODGE, France) was given.

Using standard aseptic techniques, a cutaneous incision was made on the medial face of the knee. The medial condyle of the stifle joint of each animal was exposed by blunt dissection of the subcutaneous tissue and the fascia. The muscle and joint capsule were gently dissected and the supracondyle bone area was approached. The periosteum was carefully removed from the femoral epiphysis and metaphysis to expose the implant site. A core drill was used to drill a 10 mm diameter × 15 mm deep hole in the distal femoral epiphysis. After drilling the defect to the required depth, an extractor was inserted and driven down to the bottom of the defect. The extractor was turned to break the bone trabecula at the bottom of the defect. Depth of the defect was measured. Each defect was filled with either the test or the control article. The incision was then closed by closing the subcutaneous layers one by one with absorbable sutures (Vicryl 2-0 and Vicryl 1, ETHICON, France), and the skin layer was closed using surgical staples. The animal was repositioned and the other bone replacement material implanted in the same fashion. At the appropriate termination interval, the designated sheep were terminated by lethal injection of barbiturate (DOLETHALND, VETOQUINOL, France). A macroscopic examination of the implanted femoral epiphyses was performed. The epiphyses were then harvested and identified. The epiphyses from the 2 and 3 months animals were placed in 10% buffered formalin. After fixation, these samples were submitted for μ-CT analysis. The epiphyses from the 3 months interval were arbitrarily divided so that sixteen sites sampled from eight animals (eight test and eight control) were selected for biomechanical testing and the remaining sites were fixed in 10% buffered formalin.

#### Preparation of the samples

**Figure 2:**
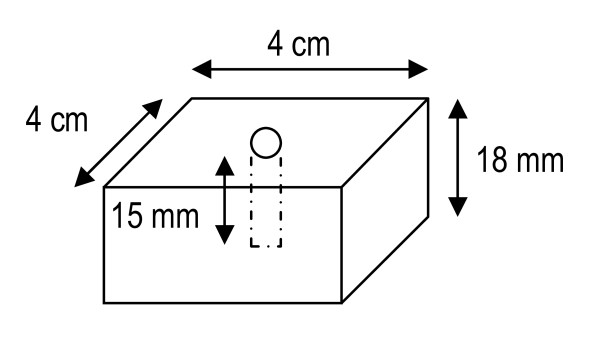
**Ostim^® ^agglomerates in transmission electron microscopy**.

The epiphysis from the eight designated sheep from the 3 months termination interval was subjected to biomechanical testing. The soft tissues were removed from these samples. The defect was located using X-rays. A diamond saw was used to obtain two parallel and flat surfaces. The approximate final dimensions of the sample were 4 × 4 × 1.8 cm (see fig 2). Care was taken not to disrupt the implant from the defect site at this stage.

The test specimens were prepared so that the implantation site were in the centre of the sample. The tissue samples were provided in physiological serum and the trials were performed within 48 h after removal. Their thickness has been measured using a slide caliper at the implantation point.

#### Biomechanical testing

First, the thickness of the samples was measured with a calliper. Following thickness measurements, the samples were immersed in room temperature saline solution. The samples were submitted to an indentation test. Load was applied axially to the specimens by a pin with a diameter of 4 mm and with a displacement speed of 0.4 mm/min until failure. To determine the mechanical characteristics of the samples, the data collected were stiffness and Young's modulus, yield point, maximum load and corresponding stress and strain. This biomechanical testing was also performed on four non implanted test articles.

Non implanted samples were prepared by placing Ostim samples in a cylindrical container of a diameter of 12 mm and a height of 20 mm. They were tested immediately thereafter.

#### μ-CT analysis

Following fixation, the epiphyses sites for the 2 months animals and the ten animals designated from the 3 months interval were used to quantify the mean BV/TV (Bone + substitute volume/Total bone defect volume). The μCT measurements were conducted at ETH Zürich by means of a μCT 80, Scanco Medical AG, Switzerland. The measured data was filtered using a constrained Gaissian filter with finite filter support (1 Voxel) and filter width (σ = 1.2). The images were then binarized to separate the object from the background using a global thresholding procedure. For density analysis a cylinder of 11.7 mm in diameter and 17 mm length was analyzed. The cylinder dimension was obtained from the μCT images and was chosen to represent the original defect as good as possible. For this the cylinder was morphed along the axis of the defect zone in the following way: A circle with 11.7 mm was positioned over the defect zone on the first and the last μCT slice of the given range of the substitute (17 mm) and the cylinder was morphed in-between. Within this cylinder the apparent density was computed as the ratio of substitute versus background.

#### Histopathological technique

Each implant site and surrounding tissue was isolated using a band saw. After labelling and fixing in 10% buffered formalin solution, the sites were longitudinally divided in two blocks. One half of each site was dehydrated in alcohol solutions of increasing concentrations, cleared in xylene, decalcified and embedded in paraffin. Embedded sites were then longitudinally cut at 5 to 7 μm using a microtome (MICROM^®^, France). One paraffin section per site was prepared and stained with Masson's trichrome. The other half of each site was dehydrated in alcohol solutions at increasing concentrations, cleared in xylene and embedded in polymethylmethacrylate (PMMA). One longitudinal section was prepared using the Exakt system [25,50]. The resin sections were stained with a modified Paragon.

#### Qualitative evaluation of implanted sites

Histological sections were observed using a Nikon microscope (Eclipse E600) fitted with ×4, ×10, ×25 and ×40 objectives. Semi-quantitative analysis was performed in accordance with ISO 10993-6 standard: local tolerance criteria and performance were analyzed and graded. (necrosis, fibrinous exudate, tissue degeneration, inflammatory cells, fibrous tissue, osteolysis, newly formed bone, osteointegration, osteoconduction, and material degradation.)

Particular attention was devoted to:

- the neoformed bone, the direct bone/implant interface and the osteoconduction process

- inflammatory reaction,

- presence of fibrosis

- material degradation.

The semi-quantitative scoring system is shown in additional file 2

#### Quantitative histomorphometry of implanted sites

The histological slides obtained from resin blocks were evaluated under a Zeiss Axioscope microscope fitted with ×5, ×10, ×20 and ×40 objectives and equipped with a color image analyzing system SAMBA^® ^(SAMBA TECHNOLOGIES, France). Two standard areas of investigation were located peripherally to the Bone/Implant junction, and two standard areas of investigation were located internally to the Bone/Implant junction.

In these areas of investigation, whenever possible, the following parameters were measured in the control resin slides and in the test resin slides at one, two and three months:

- bone to implant contact, BIC (expressed in percentage)

- bone density around the material (expressed in percentage)

- bone density inside the material (expressed in percentage)

At each time point a statistical analysis (ANOVA method) was performed for these 3 parameters, considering control versus test article.

#### Statistical analysis

The statistical tests used for a comparison between groups for the main parameters of biomechanical testing and quantitative histological analysis were Mann and Whitney or ANOVA tests performed with a software (SPSS^® ^version 14.0, edited by SPSS Inc, Chicago).

## Results

### Biomechanical testing

Biomechanical testing was performed on 16 samples from the 3 month time-period (see additional file 3). At yield point, the stress was higher in the Alpha-BSM^® ^Group and the strain was higher in the Ostim^® ^group. Resistance, stress and strain were higher in the Alpha-BSM^® ^group. Elasticity, stiffness and Young modulus were higher in the Ostim^® ^group. However, no statistically significant differences were observed for any parameters. The biomechanical testing performed on four non implanted test articles showed a maximum load for resistance and yield point at least ten times lower than the values observed after implantation, suggesting a significant hardening and/or bone ingrowth during the 3 month implantation period.

### μCT analysis

**Figure 3:**
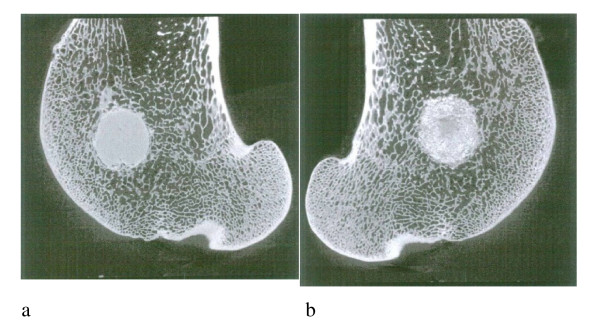
**Defects centralized, confined (a) Alpha-BSM^® ^and (b) Ostim^®^**. The μCT images after three months of the bone replacement material implantation.

All samples were successfully scanned with μCT. From these measurements it can be seen that the Alpha BSM material was very dense and had a very well confined shape. For this reason, the median for these groups was higher than 95%, which means that there was almost no porosity. The mean BV/TV (Bone + substitute volume/Total volume) values were found to be 95.2% for the 2 month Alpha BSM sites and 90.4% for the 3 month Alpha BSM sites. The Ostim material was in most samples very granular and showed some tendency to penetrate into the surrounding area. However, bone ingrowth into the substitute could not be determined as it was not possible to automatically differentiate the bone from the substitute due to similar contrast. In samples where the substitute disappeared from the original defect, there was only little bone formation that could be seen in the defect. The apparent density in the Ostim sites was lower than in Alpha BSM sites. The mean BV/TV values were found to be 68.3% for the 2 month Ostim sites and 79.9% for the 3 month Ostim sites. The relatively large range indicates that these samples were much more inhomogeneous as the control samples. The μCT measurement were limited by the fact that the bone replacement materials had a very similar attenuation for X-rays as bone and that thus two phases could not be separated automatically. It was observed that the Ostim article seemed to diffuse easily in particular when the defects were created in a spongious bone area, close to the diaphysis or the joint-space narrowing whereas the more compact Alpha BSM article used in similar conditions stayed in the defect. This led to a lower apparent density within the defects treated with the Ostim when compared to the Alpha BSM (see fig. 3a - b).

Microscopic analysis of femoral sites at 1 month (see additional file 4)

### Alpha BSM

**Figure 4:**
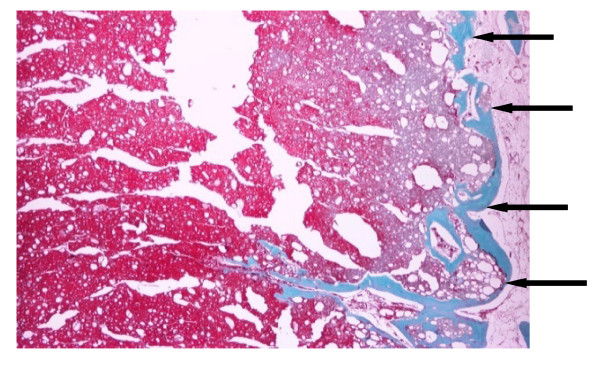
**Alpha-BSM^® ^at 1 month**. Masson's tricrome staining and 2× magnification of decalcified bone with calcium phosphate appearing as a still relatively compact matter. The osteoconduction process and newly formed bone were of limited grade and localized onto the interface (black arrows) between the edge of the original bone defect and the implant surface. (New bone = blue, Alpha-BSM = red)

All ten implanted sites were identified on the histological sections. These sites were filled with a dense green-red stained biomaterial (on paraffin sections) with diffuse optically empty round cavities. On resin sections the material was very dense, dark pink colored and it showed no signs of open porosity. Limited signs of inflammatory reaction were visible in the area surrounding the product and were accompanied by the presence of macrophages and giant multinucleated cells. The Alpha-BSM showed good signs of osseointegration. A slight grade of fibroconnective tissue was often observed at the bone-implant interface. Almost no signs of implant degradation were visible. Few osteoblasts were observed directly on the surface of the implant. The overall osteoconduction process and newly formed bone were of limited grade and localized onto the interface between the edge of the original bone defect and the implant surface. No or very limited signs of bone ingrowth within the implant were observed, likely due to the absence of open porosity of the implant. As a consequence the bone density within the implant was of very limited grade (see fig. 4)

### Ostim^®^

**Figure 5:**
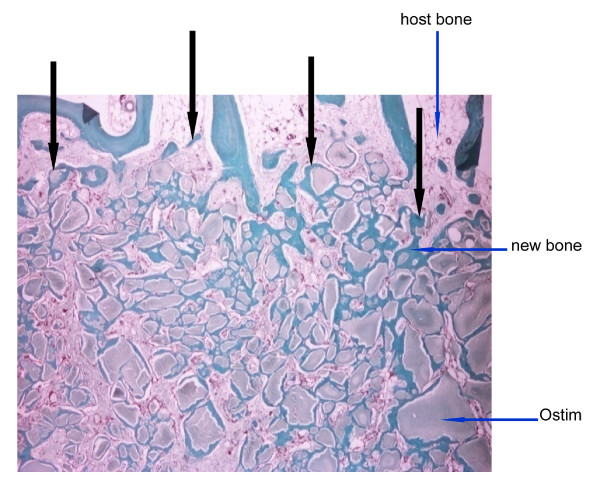
**Ostim^® ^at 1 month**. Masson's tricrome staining and 2× magnification of decalcified bone 1 month after Ostim implantation. Particles of the HA-nanocrystalline paste are seen as round structures of different size in the bone marrow and within the bone trabecular tissue. The interface between implant material and host bone is indicated by the black arrows.

Ten implant sites were identified. The implant was recognized as green stained particles (on paraffin sections) of various sizes ranging from a few μm to 100-200 μm. In resin sections, the implant particles often formed central dense and peripheral loose aggregates, thus delimiting widely open interconnected peripheral porosities. No continuity with the neighboring articular cartilage could be observed microscopically on both paraffin and resin sections. A slight to moderate grade of inflammatory reaction was observed in the area surrounding the implant particles and was accompanied by the presence of a vascularized fibroconnective inflammatory tissue, macrophages and multinucleated giant cells. The Ostim^® ^35 bone substitute showed good signs of osseointegration (moderate grade), osteoconduction (moderate grade) and bone density within the implant (slight to moderate grade). Notable was a degree of heterogeneity was observed amongst the animals regarding new bone formation and bone density. Limited signs of material degradation were observed (see fig. 5).

Microscopic analysis of femoral sites at 2 months (see additional file 4)

### Alpha BSM

**Figure 6:**
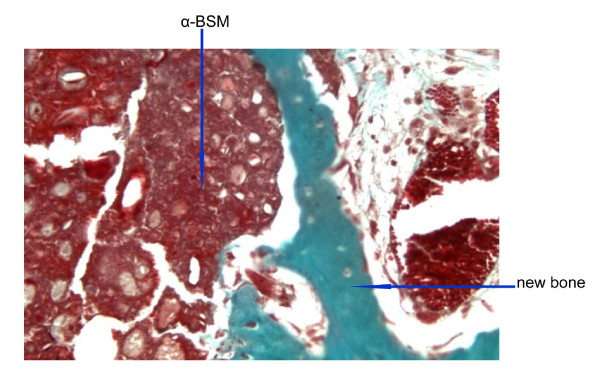
**Alpha-BSM at 2 months**. Masson's tricrome staining and 20× magnification of decalcified bone 2 months after Alpha-BSM^® ^implantation.

The ten sites implanted with Alpha BSM showed a similar appearance compared to those observed at 1 month, with only slight signs of peripheral degradation after 2 months. Almost no residual signs of inflammatory reaction were visible: no biologically significant amounts of macrophages, giant multinucleated cells and fibroconnective tissue were observed. The osseointegration of the Alpha-BSM^® ^slightly progressed with few signs of penetration of thin bone trabecules within the outer portion of the bone substitute. Few signs of bone remodeling were observed (see fig. 6)

### Ostim^®^

**Figure 7:**
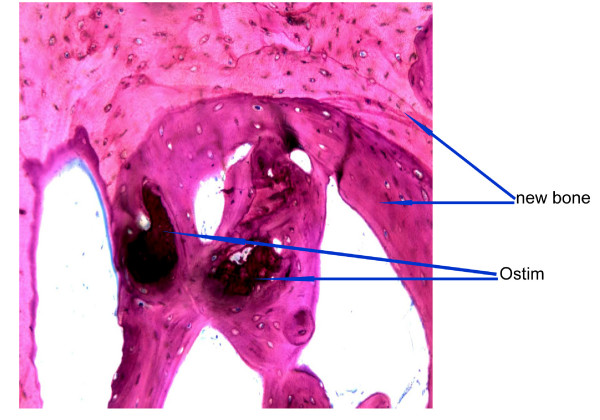
**Ostim^® ^at 2 months**. Modified paragon staining and 20× magnification of Ostim^® ^core surrounded by new bone formation.

The overall aspects of Ostim^® ^after 2 months were comparable to those observed at 1 month, with some sites showing a heterogeneous structure of the material. The degradation process of the test article progressed significantly between 1 and 2 months with an overall reduced size of the particles in many animals. No continuity with the neighboring articular cartilage could be observed microscopically on both paraffin and resin sections. Ostim^® ^particles were associated with few signs of inflammatory reaction consisting of a limited grade of macrophages and giant multinucleated cells. A variable grade of fibrocytic tissue was visible within the implanted bone defect. Mean osteoconduction and bone density parameters progressed very significantly. Few signs of bone remodeling were observed (see fig. 7).

Microscopic analysis of femoral sites at 3 months (see additional file 4)

### Alpha-BSM^®^

**Figure 8:**
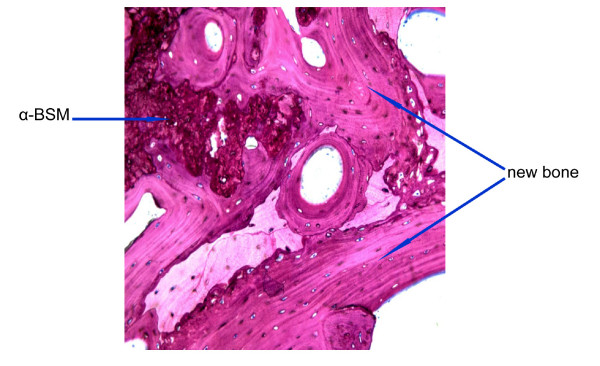
**Alpha-BSM^® ^at 3 months**. Modified paragon staining and 10× magnification of Alpha BSM^® ^cement on the left upper quadrant of the image surrounded by newly formed bone.

Ten implanted sites were identified on the histological sections. In paraffin sections the sites were filled with a dense red stained biomaterial with diffuse optically empty round cavities. In resin sections, the material was still quite dense, but some signs of open porosity were observed. Limited signs of inflammatory reaction were visible in the area surrounding the product and were accompanied by the presence of macrophages. Moderate evidences of bone ingrowth within the implant were observed. As a consequence the bone density within and around the implant was of slight to moderate grade, and a steady and homogeneous progression of new bone formation was observed amongst the animals in resin sections. Some signs of bone remodeling were observed in one animal only on resin sections (see fig. 8)

### Ostim^®^

**Figure 9:**
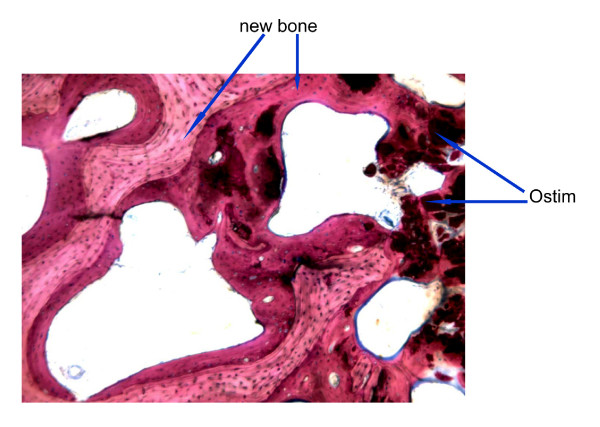
**Ostim^® ^at 3 months**. Modified paragon staining and 5× magnification of non-decalcified bone 3 months after Ostim^® ^implantation. Ostim^® ^bone showed good osseointegration and bone remodeling.

The overall histopathological picture was very similar to the one observed at 2 months. The mean amount of inflammatory reaction surrounding the implant particles was slight and tended to decrease over time. Still there were great individual variations regarding the amount of fibrous tissue ranging from absent to marked grade, resulting in respectively high or low bone density. Ostim^® ^showed good signs of mean osseointegration, osteoconduction and bone density within the implant that improved over time. Moderate signs of material degradation were constantly observed, which increased over time. Some signs of bone remodeling were observed in resin sections (see fig. 9).

### Quantitative histolomorphometrical analysis

At early time points, Ostim^® ^showed much better microscopic signs of osteointegration, osteoconduction and overall bone ingrowth when compared to the Alpha-BSM^®^. After 2 and 3 months, the bone regeneration level within the Ostim^® ^treated sites was better than the Alpha-BSM^® ^treated sites, but new bone formation tended to slow down when compared to the steadier progression and fewer inter-individual variations observed with Alpha-BSM^® ^(see additional file 5 and 6).

### Bone to Implant Contact (BIC)

After 1 month, bone to implant contact was significantly different between alpha BSM and Ostim groups (p = 0.004). Bone to implant contact around the material was found higher in the alpha BSM than in the Ostim group. This difference was transient and not observed at later time-points (see additional file 7).

### Bone density inside the material

**Figure 10:**
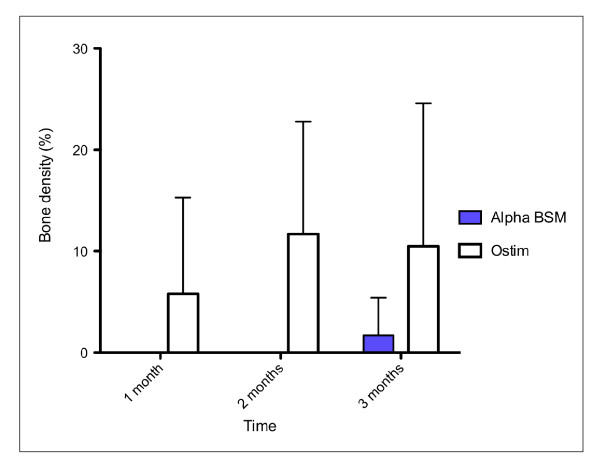
**Bone density inside the material in percent**. Bone ingrowth was detected only 3 months postoperatively with alpha BSM a bone substitute.

After 1 and 2 months, bone density inside the material was significantly different between Alpha-BSM^® ^and Ostim^® ^groups. Bone density was higher in Ostim^® ^group when compared to Alpha-BSM^® ^as no bone was found inside the Alpha-BSM^® ^material. After 3 months, this same parameter was again significantly higher in the Ostim^® ^group as compared to the Alpha-BSM^® ^group (p = 0.012) although some bone was found inside the Alpha-BSM^® ^material (see fig. 10).

Bone density around the material did not show statistically significant variations at all considered time-points.

## Discussion and Conclusions

There were a number of attempts in the past to replace bone loss with bone replacement materials. Hamilton in 1881 used disinfected sponges and Gluck in 1891 implanted ivory into bone defects. Dreesmann even tried gypsum in 1892. In the 20th century Maatz experimented with bovine bone and Tarsoly filled the defects with egg shells[51]. Modern bone replacement materials composed of calcium phosphate are either extracted from bovine cancellous bone or are produced synthetically[14,18,52,53]. The requirements of an ideal bone replacement material have not changed over the past 30 years: simple application, osteoconductive properties, and complete resobarbility during bone ingrowth[53,54].

Alpha-BSM^® ^fulfils these requirements very well as a synthetic calcium phosphate bone replacement material. Compared to other calcium phosphates which require five years or more to be resorbed by the host bone[55,56], Alpha BSM^® ^appears to undergo more rapid resorption[22,52]. It does, however, have less compressive strength than other types of cements [34]. Compared to autologous cancellous bone, calcium phosphate cements have shown superior compressive strength. Welch et al reported a significantly lower secondary loss of correction with the use of Alpha-BSM^®^, in the treatment of tibia head fractures in goats compared to autologous spongiosa[34]. Russell also revealed a significantly lower subsidence in tibia head fractures with the use of Alpha-BSM^® ^compared to autologous bone grafts in the scope of a multicentre study with 119 Patients[39].

The cement microstructure of Alpha-BSM^®^, which results from a reaction of precipitation, is not equivalent to the crystalline structure of human bone[18,57,58]. The nanocrystalline structure of Ostim^® ^on the other hand resembles human bone much closer (Calcium/Posphate ratio of 1,675 and specific surface area of 106 m^2^g^-1 ^is synthesized by a wet chemical reaction of precipitation under permanent pH control using CaO dispersed in water under constant stirring to maintain a suspension state and H3PO4 as starting material [47,58]. Ostim^® ^has been observed as needle shaped HA crystals forming agglomerates in transmission electron microscopy [1,47]. According to the hypotheses put forward by Constanz and Knaack such HA-compounds accelerate the bony ingrowth into the CSD as they closely mimic the required resorptive and osseointegrative properties of poor crystalline apatitic structure of natural bone[52,57,58]. Moreover Kilian et al also presented evidence of angiogenesis within bone defects filled with nanocrystalline hydroxyapatite [59,1,60]. These properties appear to explain the significantly increased ingrowth of bone of the Ostim^® ^group compared to the Alpha-BSM^® ^group. There was no significant difference between the two materials with regard to their biomechanical stability.

Ostim^® ^35 showed limited signs of degradation after 1 month but degradation increased over time and was of moderate grade after 3 months in our experimental series. This confirms the results obtained in a number of different experimental animal models of other studies. Grigoryan et al described the rapid bone ingrowth at a low complication rate following the treatment of jaw defects with Ostim^® ^in dogs in 2000 [61]. Our own animal experiments dealing with the filling of critical size defects with Ostim in New Zealand white rabbits also resulted in swift and uniform bone ingrowth[62]. The results were further enhanced by the additional use of a sintered hydroxyapatite ceramic[62]. Ostim^® ^was always present in the bone after 60 days following implantation, as in the present study[62]. Laschke reported a guided neovascularization directed toward areas of Ostim^® ^degradation in Syrian golden hamsters [63]. Spiess further confirmed the good biocompatibility, osteoconductivity and bone ingrowth in New Zealand whites, but reported a halt in the Ostim^® ^degradation process 6 weeks following implantation[64].

Comprehensive clinical experience of using the hydrated HA-paste as a void filling exists in the field of maxillofacial surgery. Various stomatology publications describe an accelerated fracture healing and bone density increase at a high degree of tolerance. In 1996 Zuev treated 395 patients with jaw defects and peridontal abscesses. The complication rate of the 200 patients in the Ostim group was at 1.5% compared to 3.6% in the group with 195 patients treated with allografts[44]. Bezrukow achieved excellent results in 1998 after treating 49 patients with Ostim^® ^following cystectomy of benign cyst-tumours of the jaw. The defects in all 49 patients were replaced with fresh bone three months following the defect filling [43]. Gerlachfilled 44 mandibular cysts with Ostim^® ^and reported complete material resorption with an extemely low complication rate[65]. Strietzel et al performed a lateral alveolar ridge augmentation with Ostim^® ^in 14 patients. Six months later histological results showed good bone ingrowth of the defect and small amounts of Ostim^® ^remnants [46].

Ostim^® ^in combination with conventional or angularly stable osteosynthesis has also been successfully used in the field of traumatology. Particularly good results were achieved in prospective cohort studies with patients for the treatment of radial fractures, with closed intraarticular calcaneus fractures, as well as tibia compression fractures [40-42]. Histological examinations of bone harvested from patients during elective metal removal, have shown excellent bone ingrowth into the past bone defect with well structured cortical and cancellous bone tissue and without any inflammatory reaction orosteofibrosis. The implanted material was mainly resorbed[62].

Our data verifies the importance of the nanocrystalline hydroxyapatite in the treatment of metaphyseal osseous volume defects in the metaphyseal spongiosa, even though this study was done on an experimental animal model. Further clinical studies need to be conducted to validate the significance of Ostim in human bone defects. Due to its good biocompatibility and high rates of resorption we can see a number of indications for the use of Ostim in the field of traumatology. In our opinion the application of Ostim^® ^should always be combined with some form of stable osteosynthesis, preferably with an angularly stable plate, due to its lack of dimensional stability.

## Competing interests

Aap biomaterials is manufacturing and selling Ostim. Aap did finance the animal study which was performed by Biomatech. E. Dingeldein and L. Heinmann receive their salary and are employed by aap Biomaterials. E. Dingeldein holds shares in aap.

H. Cavey, J-P. Boutrand, X. Palazzi, and G. Clermont receive salary from Biomatech.

F-X. Huber and N. McArthur have no competing interests.

None of the authors holds or is applying for patents related to the content of the manuscript.

## Authors' contributions

F-XH and NM carried out the data analysis and drafted the final manuscript. LH and ED participated in the development of Ostim and drafted the basic study design. HC, XP and GC conducted the animal experiments and prepared the samples for further analysis. J-PB participated in further study design and coordination and statistical analysis. All authors read and approved the final manuscript.

## Pre-publication history

The pre-publication history for this paper can be accessed here:

http://www.biomedcentral.com/1471-2474/10/164/prepub

## Supplementary Material

Additional file 1 **Study design**. Number of sheep, number of investigation sites and analyses performed. Click here for file

Additional file 2 **Semi-quantitative scoring system**. The table shows how each score was correlated to various degrees of reaction. Click here for file

Additional file 3 **Results of biomechanical testing**. Geometry, Elasticity Yield point and Resistance were tested for Ostim and Alpha-BSM. Click here for file

Additional file 4 **Mean values of the semi-quantitative analysis**. A number of histological parameters have been compared between Ostim and Alpha-BSM at 1, 2 and 3 months. Click here for file

Additional file 5 **Mean histomorphometric values around the material**. Values in % are shown for both Ostim and Alpha-BSM concerning fibrous to implant contact, bone to implant contact, implant density, fibrous density and bone density around the implant. Click here for file

Additional file 6 **Mean histomorphometric values inside the material**. Values in % are shown for both Ostim and Alpha-BSM concerning fibrous to implant contact, bone to implant contact, implant density, fibrous density and bone density inside the implant. Click here for file

Additional file 7 **Significant differences between Ostim and Alpha-BSM**. The table shows that only bone density inside material remained significantly different between Ostim and Alpha-BSM throughout the 3 month study period. Click here for file
