# Supplementary material for: Evaluation of a novel nanocrystalline hydroxyapatite paste Ostim® in comparison to Alpha-BSM® - more bone ingrowth inside the implanted material with Ostim® compared to Alpha BSM®
Source: BMC Musculoskelet Disord. 2009 Dec 22;10:164. doi: 10.1186/1471-2474-10-164 (PMC2807853; doi:10.1186/1471-2474-10-164)
Supplement: Additional file 1 — Study design. Number of sheep, number of investigation sites and analyses performed. [file 1471-2474-10-164-S1.DOCX]

Additional file 1

Study design

| Time-period | Number of sheep | Number of sites per article | | Total number of sites | Analysis | | |
| --- | --- | --- | --- | --- | --- | --- | --- |
|  |  | Ostim® | Alpha-BSM® |  | Histology | µCT | Biomechanical  Testing |
| 1 month | 10 | 10 | 10 | 20 | 20 | 0 | 0 |
| 2 months | 10 | 10 | 10 | 20 | 20 | 20 | 0 |
| 3 months | 18 | 18 | 18 | 36 | 20 | 20 | 16 |
