# Supplementary material for: Evaluation of a novel nanocrystalline hydroxyapatite paste Ostim® in comparison to Alpha-BSM® - more bone ingrowth inside the implanted material with Ostim® compared to Alpha BSM®
Source: BMC Musculoskelet Disord. 2009 Dec 22;10:164. doi: 10.1186/1471-2474-10-164 (PMC2807853; doi:10.1186/1471-2474-10-164)
Supplement: Additional file 2 — Semi-quantitative scoring system. The table shows how each score was correlated to various degrees of reaction. [file 1471-2474-10-164-S2.DOCX]

Additional file 2

Semi-quantitative scoring system

| **Reaction** | **Grade** |
| --- | --- |
| None | 0 |
| Light | 1 |
| Moderate | 2 |
| Marked | 3 |
| Severe | 4 |
