# Supplementary material for: Evaluation of a novel nanocrystalline hydroxyapatite paste Ostim® in comparison to Alpha-BSM® - more bone ingrowth inside the implanted material with Ostim® compared to Alpha BSM®
Source: BMC Musculoskelet Disord. 2009 Dec 22;10:164. doi: 10.1186/1471-2474-10-164 (PMC2807853; doi:10.1186/1471-2474-10-164)
Supplement: Additional file 3 — Results of biomechanical testing. Geometry, Elasticity Yield point and Resistance were tested for Ostim and Alpha-BSM. [file 1471-2474-10-164-S3.DOCX]

Additional file 3

Biomechanical testing

| **Article** | **Geometry** | **Elasticity** | | **Yield point** | | | | **Resistance** | | | |
| --- | --- | --- | --- | --- | --- | --- | --- | --- | --- | --- | --- |
|  | **Thickness**  **e (mm)** | **Stiffness**  **K (N/mm)** | **Young**  **modulus**  **E (MPa)** | **Load**  **(N)** | **Displacement**  **(mm)** | **Stress**  **(MPa)** | **Strain**  **(%)** | **Maximum**  **Load (N)** | **Displacement**  **to maximum**  **load (mm)** | **Stress**  **(MPa)** | **Strain**  **(%)** |
| **Alpha-BSM® Mean** | 26.13 | 421 | 862 | 368 | 0.994 | 29 | 3.92 | 493 | 1.425 | 39 | 5.63 |
| **SD** | 1.51 | 193 | 351 | 425 | 0.565 | 34 | 2.42 | 465 | 0.468 | 37 | 2.09 |
| **Ostim® 18 Mean** | 26.20 | 461 | 941 | 344 | 1.203 | 27 | 4.61 | 376 | 1.368 | 30 | 5.24 |
| **SD** | 1.14 | 240 | 469 | 179 | 0.493 | 14 | 1.89 | 188 | 0.421 | 15 | 1.59 |
| **Non implanted Ostim sample 1** | 27.00 | 1.75 | 3.75 | 0.38 | 0.278 | 0.03 | 1.03 | 2.16 | 3.5 | 0.172 | 12.96 |
| **Non implanted Ostim**  **sample 2** | 27.00 | 1.96 | 4.20 | 0.43 | 0.290 | 0.03 | 1.07 | 3.08 | 3.5 | 0.245 | 12.96 |
| **Non implanted Ostim sample 3** | 27.00 | 1.77 | 3.79 | 0.65 | 0.437 | 0.05 | 1.62 | 3.57 | 3.5 | 0.284 | 12.96 |
| **Non implanted Ostim**  **sample 4** | 27.00 | 1.14 | 2.45 | 0.59 | 0.523 | 0.05 | 1.94 | 2.38 | 3.5 | 0.189 | 12.96 |
