# Supplementary material for: Evaluation of a novel nanocrystalline hydroxyapatite paste Ostim® in comparison to Alpha-BSM® - more bone ingrowth inside the implanted material with Ostim® compared to Alpha BSM®
Source: BMC Musculoskelet Disord. 2009 Dec 22;10:164. doi: 10.1186/1471-2474-10-164 (PMC2807853; doi:10.1186/1471-2474-10-164)
Supplement: Additional file 4 — Mean values of the semi-quantitative analysis. A number of histological parameters have been compared between Ostim and Alpha-BSM at 1, 2 and 3 months. [file 1471-2474-10-164-S4.DOCX]

Additional file 4

Mean values of the semi-quantitative analysis

| **Time period** | **Material** | **Infection signs** | **Fibrinous exudate** | **Necrosis** | **Tissue degeneration** | **PMN cells** | **Lymphocytes (if distinguishable)** | **Macrophages (if distinguishable)** | **Giant cells/osteoclastic cells** | **Osteoblastic cells** | **Fibrocytes and fibroconnective tissue** | **Osteolysis** | **Osteointegration** | **Bone density** | **Material degradation** | **Implant migration** | **Signs of bone remodeling** | **Osteoconduction/newly formed bone** |
| --- | --- | --- | --- | --- | --- | --- | --- | --- | --- | --- | --- | --- | --- | --- | --- | --- | --- | --- |
| **1 month** | **Alpha**  **BSM** | 0 | 0 | 0 | 0 | 0 | 0 | 1 | 0.6 | 0.3 | 1.1 | 0 | 1 | 0.7 | 0.1 | 0 | 0 | 1 |
|  | **Ostim®** | 0 | 0 | 0 | 0 | 0 | 0 | 1.4 | 1.5 | 1 | 0.9 | 0 | 1.8 | 1.8 | 1 | 0 | 0 | 1.8 |
| **2 month** | **Alpha**  **BSM** | 0 | 0 | 0 | 0 | 0 | 0 | 0.3 | 0.2 | 1.3 | 0.4 | 0 | 1.5 | 1.2 | 0.6 | 0 | 0 | 1.5 |
|  | **Ostim®** | 0 | 0 | 0 | 0 | 0 | 0 | 0.6 | 1 | 1.8 | 1.1 | 0 | 2.7 | 2.5 | 1.9 | 0 | 0 | 2.8 |
| **3 month** | **Alpha BSM** | 0 | 0.1 | 0.3 | 0 | 0 | 0 | 1 | 0 | 0.9 | 1 | 0 | 1.4 | 1 | 0.3 | 0 | 0 | 1.2 |
|  | **Ostim®** | 0 | 0 | 0 | 0 | 0 | 0.1 | 0.8 | 1 | 1.1 | 1.1 | 0 | 3.2 | 2.4 | 2.1 | 0 | 0.3 | 3 |

These scoring represents a synthesis obtained from both paraffin and resin-embedded tissues.
