# Supplementary material for: Evaluation of a novel nanocrystalline hydroxyapatite paste Ostim® in comparison to Alpha-BSM® - more bone ingrowth inside the implanted material with Ostim® compared to Alpha BSM®
Source: BMC Musculoskelet Disord. 2009 Dec 22;10:164. doi: 10.1186/1471-2474-10-164 (PMC2807853; doi:10.1186/1471-2474-10-164)
Supplement: Additional file 5 — Mean histomorphometric values around the material. Values in % are shown for both Ostim and Alpha-BSM concerning fibrous to implant contact, bone to implant contact, implant density, fibrous density and bone density around the implant. [file 1471-2474-10-164-S5.DOCX]

Additional file 5a

Mean Histomorphometric values (%) around the material

| Time period | Group | Fibrous to implant contact (%) | Bone to implant contact (%) | Implant density (%) | Fibrous density (%) | Bone density (%) |
| --- | --- | --- | --- | --- | --- | --- |
| 1 month | Alpha BSM | 48.9 | 51.1 | 20.0 | 48.3 | 31.7 |
|  | *SD* | *33.3* | *33.3* | *8.7* | *18.0* | *16.2* |
|  | OSTIM | 78.4 | 21.6 | 14.6 | 53.4 | 32.0 |
|  | *SD* | *23.4* | *23.4* | *8.8* | *11.4* | *8.7* |
| 2 months | Alpha BSM | 30.8 | 69.2 | 22.6 | 43.7 | 33.8 |
|  | *SD* | *25.2* | *25.2* | *7.4* | *11.4* | *12.1* |
|  | Ostim | 21.0 | 79.0 | 10.3 | 48.4 | 41.3 |
|  | *SD* | *23.9* | *23.9* | *5.7* | *10.5* | *10.4* |
| 3 months | Alpha BSM | 31.1 | 68.9 | 19.4 | 39.7 | 40.9 |
|  | *SD* | *11.4* | *11.4* | *8.1* | *10.4* | *10.4* |
|  | Ostim | 24.3 | 75.7 | 10.6 | 52.1 | 37.2 |
|  | *SD* | *14.3* | *14.3* | *5.9* | *16.0* | *17.6* |
