# Supplementary material for: Evaluation of a novel nanocrystalline hydroxyapatite paste Ostim® in comparison to Alpha-BSM® - more bone ingrowth inside the implanted material with Ostim® compared to Alpha BSM®
Source: BMC Musculoskelet Disord. 2009 Dec 22;10:164. doi: 10.1186/1471-2474-10-164 (PMC2807853; doi:10.1186/1471-2474-10-164)
Supplement: Additional file 6 — Mean histomorphometric values inside the material. Values in % are shown for both Ostim and Alpha-BSM concerning fibrous to implant contact, bone to implant contact, implant density, fibrous density and bone density inside the implant. [file 1471-2474-10-164-S6.DOCX]

Additional file 5b

Mean Histomorphometric values (%) inside the material

| Time period | Group | Fibrous to implant contact (%) | Bone to implant contact (%) | Implant density (%) | Fibrous density (%) | Bone density (%) |
| --- | --- | --- | --- | --- | --- | --- |
| 1 month | Alpha BSM | / | / | / | / | 0.0 |
|  | *SD* | */* | */* | */* | */* | *0.0* |
|  | Ostim | 66.9 | 33.1 | 43.2 | 45.1 | 5.8 |
|  | *SD* | *29.1* | *29.1* | *18.8* | *18.1* | *9.5* |
| 2 months | Alpha BSM | / | / | / | / | 0.0 |
|  | *SD* | */* | */* | */* | */* | *0.0* |
|  | OStim | 42.2 | 57.8 | 48.3 | 37.9 | 11.7 |
|  | *SD* | *32.0* | *32.0* | *21.5* | *16.4* | *11.1* |
| 3 months | Alpha BSM | 45.2 | 54.8 | 76.3 | 15.2 | 1.7 |
|  | *SD* | *16.8* | *16.8* | *12.4* | *11.8* | *3.7* |
|  | Ostim | 34.3 | 65.7 | 51.5 | 33.5 | 10.5 |
|  | *SD* | *25.6* | *25.6* | *23.3* | *16.3* | *14.1* |
