# Supplementary material for: Evaluation of a novel nanocrystalline hydroxyapatite paste Ostim® in comparison to Alpha-BSM® - more bone ingrowth inside the implanted material with Ostim® compared to Alpha BSM®
Source: BMC Musculoskelet Disord. 2009 Dec 22;10:164. doi: 10.1186/1471-2474-10-164 (PMC2807853; doi:10.1186/1471-2474-10-164)
Supplement: Additional file 7 — Significant differences between Ostim and Alpha-BSM. The table shows that only bone density inside material remained significantly different between Ostim and Alpha-BSM throughout the 3 month study period. [file 1471-2474-10-164-S7.DOCX]

Additional file 6

Significant differences between Ostim and Alpha-BSM

| Statistical comparison | Bone to implant contact | Bone density around material | Bone density inside material |
| --- | --- | --- | --- |
| 1 month | S | NS | S |
| 2 months | NS | NS | S |
| 3 months | NS | NS | S |

NS: Not significant, S: Significant (p<0.05)
